# Natural History Museum Sound Archive I: Orthoptera: Gryllotalpidae Leach, 1815, including 3D scans of burrow casts of *Gryllotalpa gryllotalpa* (Linnaeus, 1758) and *Gryllotalpa vineae* Bennet-Clark, 1970

**DOI:** 10.3897/BDJ.3.e7442

**Published:** 2015-12-21

**Authors:** Ed Baker, Yoke-Shum Broom

**Affiliations:** ‡ The Natural History Museum, London, United Kingdom

**Keywords:** mole cricket, *
Gryllotalpa
*, bioacoustics, burrow, cast, 3D scanning

## Abstract

**Background:**

The Natural History Museum (NHM) sound archive contains recordings of Gryllotalpidae, and the NHM collection holds plaster casts of the burrows of two species. These recordings and burrows have until now not been made available through the NHM's collection database, making it hard for researchers to make use of these resources.

**New information:**

Eighteen recordings of mole crickets (three identified species) held by the NHM have been made available under open licenses via BioAcoustica. 3D scans of the burrows of *Gryllotalpa gryllotalpa* (Linnaeus, 1758) and *Gryllotalpa vineae* Bennet-Clark, 1970 have been made available via the NHM Data Portal.

## Introduction

**Figure 1.**
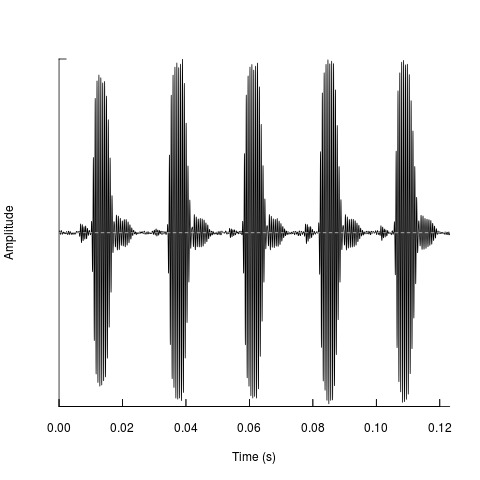
Oscillogram of *Gryllotalpa gryllotalpa*, http://bio.acousti.ca/comment/168#comment-168. The song is a continuously repating repetition of the pattern shown here. Generated by the BioAcoustica analysis tools (Baker et al. 2015).

**Figure 2.**
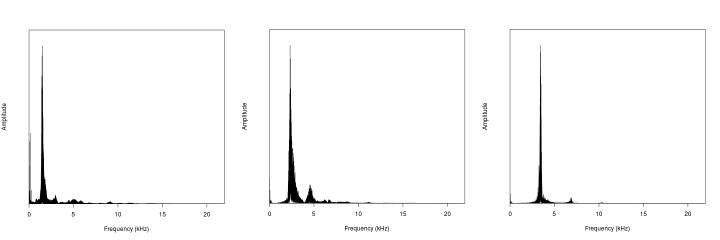
Song frequencies of (a) *Gryllotalpa gryllotalpa*, http://bio.acousti.ca/comment/48#comment-48; (b) *Gryllotalpa orientalis*, http://bio.acousti.ca/comment/51#comment-51 & (c) *Gryllotalpa vineae*, http://bio.acousti.ca/comment/64#comment-64. Generated by the BioAcoustica analysis tools (Baker et al. 2015).

Mole Crickets (Orthoptera: Gryllotalpidae) are subterranean insects, rarely seen above ground apart from adults flying during breeding season. The Natural History Museum has a collection of sound recordings and burrow casts of the genus *Gryllotalpa* Latreille 1802. The males produce a very loud song (up to 115dB in *G. vineae*:Bennet-Clark 1970a), consisting of a continuous series of syllables (Fig. 1). Compared to other orthopterans the tone is relatively pure (the frequency range varies little from the dominant frequency and its harmonics; Fig. 2). Male mole crickets sing from within their burrows, the shapes of which have been shown to closley approximate an exponential horn (Bennet-Clark 1970a). Various species are known to be pests of agriculture (e.g. Thakur et al. 2013) and turf (Walker and Nickle 1981). Other species are of conservation interest, such as *Gryllotalpa major* (Saussure, 1874) in the United States (Vaughn et al. 1993). In the UK *Gryllotalpa gryllotalpa* is restricted to a single site in the New Forest, and the latest records (from May 2014: BioAcoustica-Harrison-1) are based on its song.

The collection of Gryllotalpidae sounds held by the Natural History Museum is limited (at present there are 18 recordings of three identified species, plus unidentified material). They are presented now for the following reasons; (1) they are the only representatives of the super-family Gryllotalpoidea, and future planned publications on the Orthoptera sound collection are being written at super-family level; (2) the only burrow casts held by the NHM are of *Gryllotalpa*, and (3) the importance of *Gryllotalpa gryllotalpa* for both conservation (in the UK) and as an invasive pest (in the USA) makes publication timely.

Acoustic monitoring devices have been developed for other species of Orthoptera (e.g. Bennet et al. 2015) and studying the acoustics of Mole Crickets could therefore lead to automated monitoring for both damaging invasive species and populations at severe risk. Understanding the acoustics of Mole Crickets requires access to recordings of their songs, as well as developing an understanding of how their burrows may influence sound propagation (Bennet-Clark 1970a). This paper describes the digitised song collections and burrow cast collection of the Natural History Museum, London.

## General description

### Purpose

Online libraries of recorded wildife sound are useful in taxonomic studies (e.g. *Gryllotalpa vineae* was initially diagnosed in part based on its song, Bennet-Clark 1970b) and as reference collections for developing tools for automatic taxon identification. Recordings of Gryllotalpidae held by the Natural History Museum (NHM) have been digitised and made available on the BioAcoustica platform (Baker et al. 2015: http://bio.acousti.ca/taxonomy/term/178).

**Figure 3.**
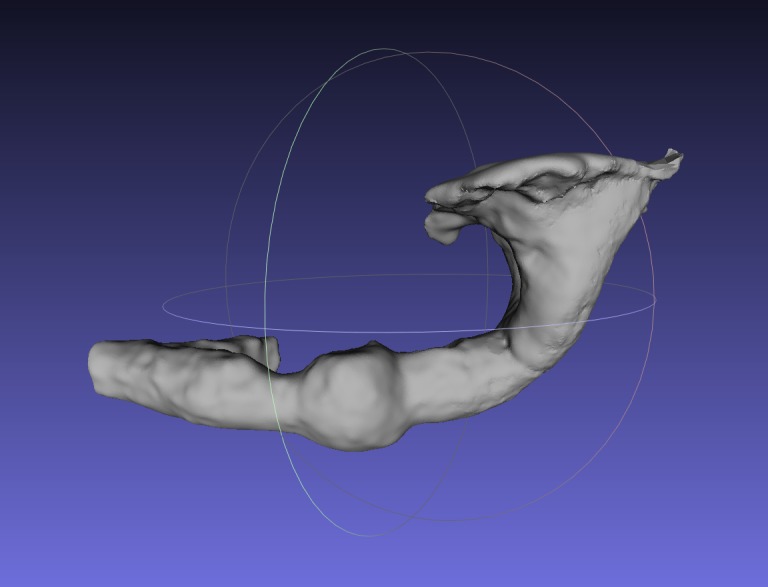
Mesh from 3D scan of the burrow of *Gryllotalpa vineae*. Viewed in Meshlab. STL file available in Baker 2015. The files are also available from the BioAcoustica Burrow Casts GitHub repository.

3D scans of burrow casts of the species *Gryllotalpa gryllotalpa* and *Gryllotalpa vineae* have been made (Figs 3, 4) and published via the NHM Data Portal (Baker 2015). These casts underpin the work of Bennet-Clark (1970a) who investigated the role of burrows in the acoustic ecology of these species. The scans were made using a NextEngine 3D Scanner HD and the accompanying ScanStudio softare. Digital 3D models of burrows could be used to model their acoustic behaviour using digital 3D sound analysis tools.

## Project description

### Title

Digitising the NHM Wildlife Sound Collection. I. Orthoptera: Gryllotalpidae.

### Funding

This work, in part, uses tools developed by the Natural History Museum Departmental Investment Fund (DIF) award SDF 14011.

## Geographic coverage

### Description

**Figure 5.**
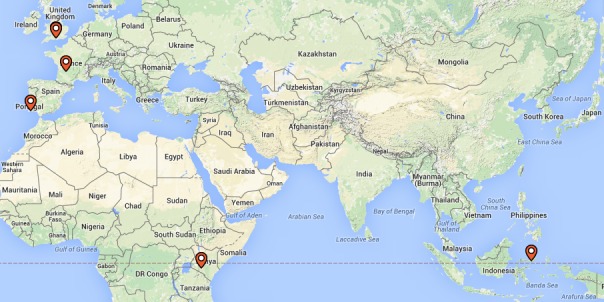
Geographic location of recordings of *Gryllotalpa* held by the Natural History Museum.

Recordings are held from Europe (*Gryllotalpa gryllotalpa*, *Gryllotalpa vineae*), Africa (Gryllotalpa cf. africana, *Gryllotalpa* sp.) and Asia (*Gryllotalpa orientalis*). Locations are summarised in Fig. 5.

## Taxonomic coverage

### Description

Recordings of three species of *Gryllotalpa* have been made available on BioAcoustica. Additionally scans of the burrow casts of two species have been made available online via the NHM Data Portal.

### Taxa included

**Table:** 

Rank	Scientific Name	Common Name
species	*Gryllotalpa gryllotalpa*	European Mole Cricket
species	*Gryllotalpa vineae*	
species	*Gryllotalpa orientalis*	

## Traits coverage

### Enter subsection title

Enter subsection text

## Usage rights

### Use license

Other

### IP rights notes

BioAcoustica uses a flexible licencing model where contributors may choose a licence for each recording they upload. Recordings owned by the Natural History Museum are available under a Creative Commons Attribution (CC BY) Licence. 3D models are made available under the Creative Commons Attribution Non-Commercial (CC BY-NC) licence following standard NHM policy. Other resources on BioAcoustica may use different licenses.

## Data resources

### Data package title

Burrow cast and sound data for NHM Gryllotalpidae

### Resource link

http://dx.doi.org/10.5519/0002120

### Number of data sets

1

### Data set 1.

#### Data set name

NHM Specimens relating to sound recordings of Gryllotalpidae

#### Data format

CSV

#### Number of columns

6

#### Download URL

http://data.nhm.ac.uk/dataset/burrow-casts-of-the-mole-cricket-genus-gryllotalpa-latreille-1802/resource/e0e36679-9a6b-4da4-8b82-eabaa6f7f147

#### Description

**Data set 1.** 

Column label	Column description
Specimen	Unique ID of the specimen (see 'NHM Specimen Idenitifers')
Type	Type of specimen of observation
Species	Species name
Link to specimen	Link to the specimen record on BioAcoustica
Link to recording	Link to recording record on BioAcoustica
Link to burrow cast	Link to burrow cast on the NHM Data Portal

## Additional information

### Gryllotalpa sp. cf. africana

The existence of a specimen (NHMUK-BMNH(E)-010210942: recording) which B. C. Townsend considered (via a specimen label) has male genitalia conspecific with *G. africana* Palisot de Beauvois, 1805, but which has a unique song may indicate the presence of cryptic species within the genus.

### Missing recordings

A recording of a specimen of *G. rufescens* Chopard, 1948 collected by W. J. Bailey and identified by B.C. Townsend cannot be located. In addition a recording of the holotype of *G. vineae* is missing from the collection. This highlights the importance of documenting sound collections and ensuring their proper care. BioAcoustica aims to address these issues by ensuring digital copies are securely backed up (as described in Baker et al. 2015) but perhaps more importantly by ensuring that collections of recorded wildlife are accessible and used. Making the collection available online is the first step towards turning the NHM Sound Archive into an actively used and developed resource.

### NHM Specimen Identifiers

Traditionally the Entomology Department of the Natural History Museum has indexed 'specimen lots' using the format 'B.M. YEAR-#' where # is a sequential number for the given year. Individual specimens were therefore rarely assigned a unique identifier (the original "B.M. numbers" are provided for specimen records in BioAcoustica). Recently the museum has moved away from the classic BMNH coden to NHMUK, while entomology specimens have been given unique identifiers of the form BMNH(E). This results in the rather long DarwinCore identifiers NHMUK-BMNH(E)-########. These specimens are currently being enterred into the Museum's collection managaement system.

For specimens where we have recordings that can be georefrenced and dated, but there is known to be no corresponding specimen in the NHM collection, the identifiers of the observation are given the format BioAcoustica-Collector-#.

## Figures and Tables

**Figure 4.** Laser scanning the burrow cast of *Gryllotalpa vineae*. This process is repeated numerous times from different angles.
